# The Effect of Aviva Exercise Intervention on Pain Level and Body Awareness in Women with Primary Dysmenorrhea

**DOI:** 10.3390/medicina60010184

**Published:** 2024-01-20

**Authors:** Zoltán Kovács, Ekine Atombosiye, Gabriella Hegyi, Henrik Szőke

**Affiliations:** 1 Department of Obstetrics, Robert Hospital, 1135 Budapest, Hungary; od4cdb@tr.pte.hu; 2 Faculty of Health Sciences, Doctoral School of Health Sciences, University of Pécs, 7621 Pécs, Hungary; gabriella.hegyi@etk.pte.hu (G.H.); henrik.szoke@etk.pte.hu (H.S.); 3 Department of Integrative Medicine, Faculty of Health Sciences, University of Pécs, 7621 Pécs, Hungary

**Keywords:** primary dysmenorrhea, pain, exercise, body awareness

## Abstract

*Background and Objective*: Primary dysmenorrhea (PD) is one of the most common clinical disorders in women of reproductive age. Our aim was to examine whether a twice-weekly thirty-minute Aviva exercise intervention could result in improvements in pain level and body awareness in patients with PD. *Materials and Methods*: In our prospective observational trial, the observation period included two consecutive menstrual cycles and the period of the next menstrual bleeding. The first menstrual bleeding period was the first measurement time (T1), the second was the second measurement time (T2), and the third was the third measurement time (T3) in a total of 78 volunteers. The primary endpoint was the change in the level of menstrual pain according to the Numeric Rating Scale (NRS) questionnaire between the intervention group (IG) and the control group (CG) at T1, T2, and T3. In this study, the secondary outcomes were the differences between the IG and CG regarding the different subscales of the Hungarian version of the Body Awareness Questionnaire (BAQ-H) at T1, T2, and T3; the Borg scale results of the IG; and adherence to the intervention. Statistical tests such as independent-sample *t*-tests, chi-square tests, Pearson’s linear correlation coefficient, and repeated-measure ANCOVA were used for the analyses. *Results*: In total, 78 volunteers were enrolled: 40 persons in the IG and 38 in the CG. There was a significant change in the level of menstruation pain according to the NRS questionnaire between the IG and CG (*p* < 0.001). There was no significant difference between the IG and CG regarding the different subscales of the BAQ-H. Only in the case of the “Note responses or changes in body process” subscale of the BAQ-H was there a trend-like effect from the Aviva exercises (*p* = 0.086). *Conclusions*: The Aviva exercise could contribute to pain relief from PD. Regarding body awareness, no significant difference was found between the two groups. Due to the short detection period and prospective observational design, our results are preliminary and need to be confirmed in larger clinical trials.

## 1. Introduction

Primary dysmenorrhea (PD) is a painful chronic condition occurring monthly with menstruation and without a definite underlying pathology [1]. Studies have highlighted this disorder as one of the most common problems among both adolescent girls and adult women of reproductive age [2]. In a systematic review and meta-analysis, Armour et al. demonstrated the significant academic impact of dysmenorrhea, with 20.1% reporting absence from school or university due to dysmenorrhea and 40.9% reporting classroom performance or concentration being negatively affected [3]. This disorder does not only decrease the quality of life [4] but also results in changes in social life and participation in physical activity. PD typically begins within three years after menarche [5]. In contrast to PD, secondary dysmenorrhea (SD) involves an underlying pathology, such as endometriosis, pelvic inflammatory disease (PID), or myoma [6].

The most important physiological factor in the development of PD is the increased level of prostaglandins in the menstrual blood [7]. In particular, PGF2α is known to stimulate uterine contractions, thereby reducing uterine circulation and causing local hypoxia [8]. In addition to prostaglandins (mainly PGE2 and PGF2α), pro-inflammatory cytokines such as TNFα also play a role in the development of dysmenorrhea [9,10,11]. A research group from Hong Kong recently showed that increasing the level of progesterone can lead to a decrease in the production of prostaglandins and pro-inflammatory cytokines and, thus, a decrease in the perception of pain [12,13]. Physical exercises are known to increase the level of endorphins and endocannabinoids in the blood [14,15]. Short-term exercise reduces cortisol production and has a non-specific analgesic effect [16].

Women seem to actively seek out and use non-pharmacological intervention therapies for relieving menstrual symptoms besides oral contraceptive pills and nonsteroidal anti-inflammatory drugs (NSAIDs) [17,18]. The definition of exercise, according to the American College of Sports Medicine, is “physical activity characterized by using planned and structured repetitive movements to increase or maintain physical fitness” [6,19]. This also incorporates mild- and moderate-intensity exercise. Systematic reviews and meta-analyses and Canadian guidelines also recommend regular exercise as a non-pharmaceutical treatment for PD [4,20,21].

In the first article on the Aviva exercise intervention method that was published, the changes in the menstrual pain level that occurred with changes in the Doppler pulsatility index of uterine arteries (UAs) were compared between an intervention group (IG) and a non-exercising control group (CG) [22]. The twice-weekly thirty-minute Aviva exercise intervention method is based on a carefully structured, mild-to-moderate intensity series of 18 movement sequences [22]. It functions in parallel with conventional medicine to support the female reproductive system, strengthen and support the normal functioning of the body, and support the occurrence of natural and spontaneous pregnancy [23,24,25]. The Aviva exercise intervention method was chosen as the subject of this research because of positive empirical experiences with it; the aim was to support this evidence with scientific research on pain levels and body awareness in women with PD for the first time.

Body awareness is an attentional focus on and awareness of internal body sensations [26]. It refers to the subjective, phenomenological aspect of proprioception and interoception that enters conscious awareness, which can be modified by mental processes, including attention, attitudes and effects, appraisal, beliefs, memories, and conditioning [26]. In psychological and medical scientific articles, definitions of body awareness have traditionally been dominated by the concern [27] that heightened body awareness must lead to somatosensory amplification and is maladaptive for clinical outcomes such as pain. However, when body awareness is defined as the ability to recognize subtle body cues, findings from numerous studies seem to contradict this traditional understanding of body awareness and suggest that body cues may be useful in the management of chronic diseases [27]. There are various publications on the relationship between body awareness, measured by the Body Awareness Questionnaire (BAQ), and pain [28,29,30,31]. Köteles developed the Hungarian version of the Body Awareness Questionnaire (BAQ-H) and evaluated it from a psychometric point of view among yoga practitioners and young adult controls. The results supported the validity and reliability of the BAQ-H [32].

In this study, the effect of the Aviva exercise on the level of menstrual pain in patients with PD was investigated. The effects of the Aviva exercise on body awareness were also examined because body awareness may play a role in the management of chronic diseases, as mentioned earlier. Since most women in Western countries lead sedentary lifestyles and have little free time, this study investigated the effects of 30 min of the easy-to-learn Aviva exercise twice a week. The hypotheses were that (1) there would be a significant difference in the level of menstruation pain between the IG and CG and that this change would be more favorable in the IG, and (2) there would be a significant difference between the IG and CG regarding the different scales of the BAQ-H.

## 2. Materials and Methods

### 2.1. Study Design and Data Collection

We performed a prospective observational trial in an outpatient clinic. Recruitment and data collection were continuously performed throughout the study period: 1 March 2019–30 June 2020.

All consecutive participants were enrolled for a period of two consecutive menstrual cycles and the period of the next menstrual bleeding.

The extent of menstrual pain was evaluated daily during menstruation by all participants of the IG and CG by completing the numeric rating scale (NRS) [6,33] electronically, where 0 means no pain and 10 means unbearable pain. In a clinometric study, the NRS was evaluated and validated as a good scale to rate PD pain [34].

There was a difference between the beginning of the data collection with questionnaires and the start of the Aviva exercises. No exercise was performed in the IG during the first menstrual bleeding period, which was the time of the first measurement (T1). At T1, the data collection of the NRS started on the first day of the menses regarding the level of menstrual pain in both groups. The pain scores of NRS were given daily during the menstrual bleeding period and averaged in the statistics. After T1 measurement, the participants of the IG performed 30 min long Aviva exercises twice a week until the end of this study, including the second and third menstrual bleeding periods. This study ended on the last day of the third menstrual bleeding period. The second menstrual bleeding period was the second measurement time (T2), and the third menstrual bleeding period was the third measurement time (T3). The NRS scale was completed daily by participants at T1, T2, and T3 in both the IG and CG. The scores of the BAQ-H were once completed by the participants of both groups at T1, T2, and T3.

The Borg scale is generally considered an accepted tool for assessing the perception of exercise intensity and has been used for many years as a self-reported method for participants in physical exercise training [35]. The Borg scale scores were self-reported by the IG participants immediately after the exercise intervention, indicating how exhausting they found the exercise. The scores of the Borg scale were completed by the participants of the IG on the first day of the T2. In the IG, the pulse was measured before and after 30 min of Aviva exercise. In the CG, pulse was also measured 30 min apart while sitting in a chair reading a book in a pleasant environment. Based on the slightly different durations of the first and second menstrual cycles and the duration of the third menstrual bleeding period, the number of exercising days slightly differed among the participants.

If the participants in the IG skipped questionnaires or exercise sessions on any day, these were marked as skipped days. If the participants in the CG skipped questionnaires on any day, these were also marked as skipped days. There were no data on the adverse effects of Aviva exercises during data collection.

The primary endpoint was the change in the level of menstrual pain according to the NRS questionnaire [6,33,34] between the IG and the CG at T1, T2, and T3. The secondary outcomes were as follows: The first was the difference between the IG and CG regarding the different scales of the BAQ-H at T1, T2, and T3. The BAQ measures attentiveness to normal, non-emotive internal bodily processes and sensations, specifically sensitivity to bodily cycles and rhythms, small changes in normal functioning, and the anticipation of bodily reactions, using 18 items scored on a 7-point Likert scale ranging from 1 (not at all true about me) to 7 (very true about me). The original version of the BAQ has four scales: “note responses or changes in body process”; “predict bodily reaction”; “sleep-wake cycle”; and “onset of illness” [36]. The BAQ-H contains the same 4 four scales as the original BAQ. After a minimal change, the BAQ-H contains 17 items of the original BAQ. In the BAQ-H, item number 10 of the original English BAQ (“I do not experience seasonal changes in my bodily functions”.) was deleted, which was the only reverse item [32]. The second secondary outcome was adherence to the intervention. The third was the Borg scale results for the IG.

### 2.2. Study Population

Entry criteria at the beginning of the study period included age between 18 and 44 years, body mass index (BMI) of 17–35, regular menstrual cycles with average duration between 21 and 35 days, and menstrual duration between 3 and 7 days. All patients of IG had to be newly introduced to the Aviva method of exercise. According to Committee Opinion No. 760 of the American College of Obstetricians and Gynecologists (ACOG) [37], the initial diagnosis for all patients presenting with PD included a medical, gynecologic, menstrual, family, and psychosocial history to confirm whether a patient had primary dysmenorrhea or symptoms characteristic of secondary dysmenorrhea via outpatient gynecological consultation. When a patient only presents with symptoms of PD, manual pelvic and vaginal ultrasound examinations are not necessary [37].

Exclusion criteria included current pregnancy, contraceptive use, pain medication use for PD, any case of secondary dysmenorrhea (e.g., uterine developmental disorders, PID, ovarian cysts, endometrial polyps, fibroids, or endometriosis), medications regularly taken for medical conditions, including endocrinological, neurological, or psychiatric diseases, already established regular or professional sport activity and prior severely traumatic life events (such as divorce, etc.) occurring within three months prior to the onset of the study. Participants who became pregnant during the course of this study and those who decided to end their participation before the end date of this study were excluded. Participants were also excluded if they failed to complete questionnaires adequately on three consecutive days or on five occasions.

Women from Hungary could volunteer to participate after being informed about study participation and its requirements.

### 2.3. Sample Size and Randomization of the Study Groups

Convenience sampling was used due to the fact that no preliminary or earlier data on the effects of a mild-to-moderate Aviva exercise intervention on consecutive menstrual pain in women with PD and body awareness were available. Furthermore, the COVID-19 pandemic caused several difficulties during our study period.

The IG and CG were non-randomized as participants self-selected under real-world conditions to be included either in the exercise intervention or the control arms of this study. Self-selection included voluntary participation in learning a new set of exercises and willingness to regularly practice in a group afterward. We also aimed to make our research flexible and realistic so that women from all age groups of the reproductive time of life and all over the country could apply to take part in the research, even traveling from remote locations to practice together. Volunteering was time-consuming, could be costly, and might not be compatible with work or family commitments for all participants. Considering the above, and also taking ethical considerations into account, we did not want to deprive volunteers of the opportunity to learn these exercise interventions with some effort.

### 2.4. Exercise Intervention Program

Upon entry, participants placed into the IG received a 4 h training session to learn Aviva exercises [23,24,25]. The Aviva exercises are different from general exercises, e.g., running, swimming, or walking. A total of 19 types of exercises with music were performed twice weekly under the supervision of trained instructors in groups in Budapest, Hungary. The presence of trained teachers guaranteed and controlled that the participants of the IG carried out the exercises correctly in an outpatient clinic. Exercise sessions lasted 30 min.

The exercises were a carefully structured [22], intense, and methodical series of movement sequences, including a 5 min warm-up exercise at the beginning and 5 min cooling-down exercises at the end of the intervention. Most of the exercises were performed in a standing position, with each exercise repeated continuously and successively. These included different side, cross, forward, and back steps; moving back and forth, and right–left; tapping; turning the upper body or the whole torso; leaning forward and back with straight legs; leaning forward and back with flexed knees; tilting to the sides; circulatory movements with arms; stretching and rotating the shoulders; shoulders and arms twisting right and left (head and hips facing forward); rising to the balls of the feet and maintaining balance; squatting-down and standing-up movements; swinging the legs forwards and to the sides. Some of the exercises were performed in a sitting position, repeating each exercise continuously and successively. These exercises were as follows: stretching legs, pulling back, then bringing legs to the side and lengthening, before switching legs and repeating the exercise; flexing feet and stretching legs forward, then lifting legs 45 degrees, holding this position while counting to 4, pulling knees to the chest, and leaning forward onto straightened legs. Some of the exercises were performed in a supine position repeating each exercise continuously and successively: lying on back, stretching legs out toward the ceiling, lowering legs, and stopping at 45 degrees to hold, then lowering legs again, stopping 5 cm from the floor, and dropping heels with a thud. Some of the exercises were performed in a prone position, repeating each exercise continuously and successively: lying face-down, squeezing glutes and lifting the right leg, holding this for a count of 4, before repeating the exercise with the left leg, and then with both legs together [22]. There was no break between the 19 exercises—the participants exercised continuously. The Aviva Method Manual, presenting each exercise in detail, is shown in Figure S1.

Subjects in the CG did not participate in any exercise intervention. The exercises were performed by the participants in the IG twice a week during the study period, regardless of the day and phase of the menstrual cycle. During the study period, women in the IG and CG were asked not to take any medication, including painkillers.

### 2.5. Statistical Methods

Based on the central limit theorem, we assumed the normality of the calculated statistics due to the sufficiently large sample size [38]. The comparison of the means of the two groups was performed using the independent-sample *t*-test, and if Levene’s test for equality of variances did not assume homoskedasticity, a robust version of the *t*-test was used. Frequencies were compared using the chi-square test. The strength of the relationship between scale-type variables was measured using Pearson’s linear correlation coefficient. For the comparison of measurements over several time periods, moderated by other variables, the repeated measures ANCOVA (with Greenhouse–Geisser correction) was used.

The results of the BAQ-H were used to create the four scales for T1, T2, and T3. The scales were constructed by averaging the corresponding questions of the questionnaire, and descriptive and reliability statistics were calculated. For the Borg scale data, exploratory data analysis was conducted as it indicated that the scores of this scale poorly discriminated against our subjects.

In each particular test, subjects with any of the included data missing were omitted. The significance level (*p*) was set at 0.05 for each test, and the analyses were performed using IBM SPSS Statistics version 25 (International Business Machines. Location: New York, NY, USA).

### 2.6. Registry

This study was registered at clinicaltrials.gov: NCT04618172.

## 3. Results

### 3.1. Study Population

**Figure 1:**
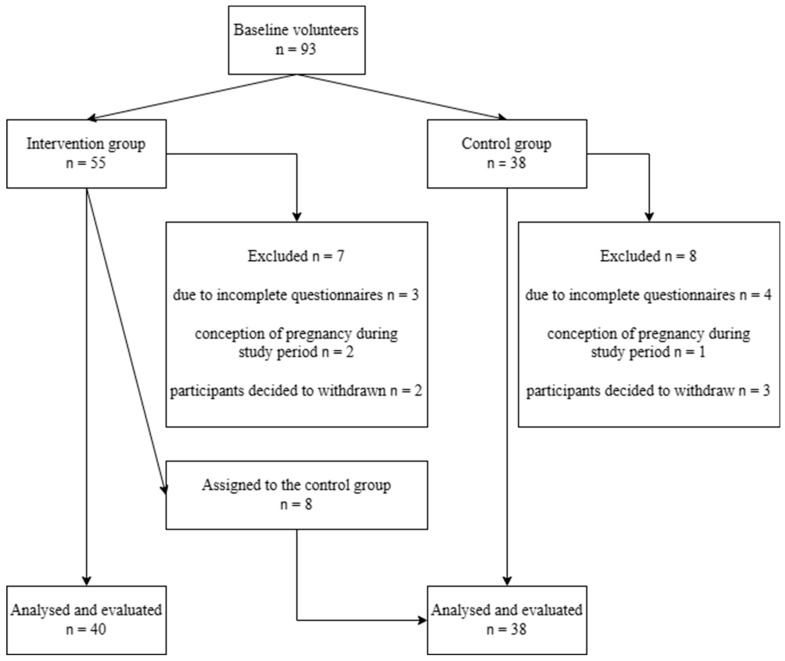
Flow chart of this study.

A power analysis was performed to demonstrate the sample size needed for specific levels of power based on partial eta squared. In total, 93 individuals volunteered to participate in the study. Due to incomplete questionnaires, three participants in the IG and four in the CG were excluded. Two participants in the IG and one in the CG became pregnant during this study and had to be excluded. Prior to the end of the study period, two participants decided to withdraw from the IG and three from the CG. Overall, a total of seven participants were excluded from the IG and eight from the CG. Eight participants (20%) of the IG spontaneously reclassified themselves into the CG. Despite signing up for the IG and agreeing to take part in the Aviva exercise intervention twice a week, they did not fulfill this condition. A total of 78 volunteers completed this study, 40 persons belonging to the IG and 38 to the CG. The flow of participants throughout this study is shown in Figure 1.

### 3.2. Distribution of Demographic Data and Dysmenorrhea Scores (NRS) at Baseline

At baseline, there were non-significant differences between the IG and CG in dysmenorrhea scores (NRS), age, BMI, duration of the first and second menstrual cycles, age at onset of menstruation, age at the first dysmenorrhea, and number of deliveries.

Demographic data and dysmenorrhea scores (NRS) at baseline in the IG and CG are shown in Table 1.

### 3.3. Primary Outcome Measures

A repeated-measure ANCOVA at T1, T2, and T3 was used to test whether the Aviva exercise had a significant effect on the change in the level of menstruation pain. In the model, in addition to the dummy variable of the Aviva exercise, participants’ age and BMI were included too.

The results show a significant difference in the level of menstrual pain according to the NRS questionnaire between the CG and IG (F(1.607, 118.907) = 12.743, *p* < 0.001, (η^2^ = 0.147). The results are shown in Table 2.

Based on the estimated marginal means of the menstrual pain of the IG and the CG at T1, T2, and T3, there was a significant decrease in pain reported in the sample among those who participated in the IG as opposed to CG (F(1.607, 118.907) = 12.743, *p* < 0.001, (η^2^ = 0.147). The results are shown in Table 3.

### 3.4. Secondary Outcome Measures

#### 3.4.1. The Difference between the IG and CG Regarding the Different Scales of the BAQ-H at T1, T2, and T3

The means of the four scales of the BAQ-H at T1, T2, and T3 were compared by using repeated measures ANCOVA with age and BMI as control variables and whether the respondent exercised twice a week using Aviva exercises. The analysis was based on the results of the repeated-measure ANCOVA of BAQ-H.

In Table 4, no significant differences were found between the IG and CG groups for any of the scales of the BAQ-H in terms of how their scores changed over the T1, T2, and T3 measurements. Only one trend-like difference can be observed for the “Note responses or changes in body process” scale: the IG group is more likely to experience a stronger increase than the CG group (F(1.823, 129.401) = 2569, *p* = 0.086, η^2^ = 0.035). A comparison of the means of the four scales of the BAQ-H of the CG and IG for the T1, T2, and T3 measurements is shown in Table 5, and the values of the trend-like difference can be seen for “Note responses or changes in body process” scale.

#### 3.4.2. Adherence to the Intervention

Eight participants (20%) of the IG spontaneously reclassified themselves into the CG. Despite signing up for the IG and agreeing to take part in the Aviva exercise intervention twice a week, they did not fulfill this condition.

#### 3.4.3. Results of the Borg Scale

According to the Borg scale, 86% of IG participants reported having experienced mild-to-moderate exertion (Borg scale: 11–14; equal to 60–75% of maximum target pulse rate) [35] after the Aviva exercise intervention. Thus, there is no significant correlation between the level of physical exertion measured on the Borg scale and the level of pain during the second menstrual period measured at T2 (Pearson’s r: 0.192, *p* = 0.202) and the level of pain during the third menstrual period measured at T3 (Pearson’s r: 0.245, *p* = 0.101).

## 4. Discussion

Despite the efficacy of NSAIDs, known as the first-line pharmacological treatment for PD, there are many adverse effects, such as gastrointestinal and neurologic reactions, which become more apparent and severe with long-term use [39]. The strategies used to manage the symptoms of PD include non-pharmacological methods, such as exercise, as well as pharmacological treatments [40,41].

The first hypothesis was confirmed. There was a significant change in the level of menstruation pain according to the NRS questionnaire between the IG and CG. This change was more favorable in the IG, which became evident at T3. There was a significant decrease in the pain reported in the sample among those who participated in the IG. Our data are in line with the results of recently published systematic reviews and meta-analyses, which recommended physical activity and exercise as an effective therapy for dysmenorrhea PD [6,42,43]. In a review of the relevant international literature, one study, similar to our own, found that exercise as little as twice-weekly reduced menstrual pain in women with PD [44]. In another study, exercise three times weekly reduced menstrual pain [45]. Other trials also have reported a reduction in menstrual pain in PD, but the type, timing of exercise interventions, durations, comparators, and outcomes and methodologies of the research significantly vary from one study to another, as published in a systematic review and meta-analysis [21,44,46].

The UAs are mainly responsible for the blood supply of the uterus [47]. The pulsatility index (PI) of UAs detected via ultrasound Doppler flowmetry measurements has become more important, as it seems to more accurately describe the blood velocity waveform [48]. Our previous research was the first in the literature to date to investigate the effect of exercise intervention on UA PI values in PD individuals [22]. The level of menstrual pain measured using the NRS and experienced by primary dysmenorrhea patients was independent of the level of blood circulation regarding the PI of the UAs [22]. The elevated PI values of UAs demonstrated reduced blood flow due to the circulatory redistribution in the participants of the IG after the Aviva exercise intervention [22]. As part of the redistribution of blood flow away from nonmuscle tissues such as the kidneys and the guts, blood flow is diverted from reproductive organs, including the uterus. The visceral redistribution of blood flow may be one way in which oxygen is conserved to fulfill the increased oxygen demands of the skeletal, cardiac, and respiratory muscles during exercise and/or part of the reflex responses required to maintain arterial pressure during high-intensity exercise [49,50]. The pain-relieving effect of regular exercise on PD manifests in other ways, not through the change in the PI of UAs. The participants of the IG performed continuous mild-to-moderate physical exercises, which relieved pain in PD through different mechanisms by altering the levels of estradiol, progesterone, endorphins, serotonin, prolactin, cortisol, and high-sensitivity C-reactive protein (HsCRP) and had a non-specific analgesic effect, as mentioned in the Introduction [12,13,14,15,16,51,52]. However, the various hormonal changes and interactions within the endocrine system that play a role in exercise-induced pain alleviation are not clearly understood [51]. An open question is whether there is a compensatory postexercise vasodilatation [53] in the PI of UAs during recovery from the exercise intervention in the IG, changing a decreased blood flow to an increased blood flow, contributing to the pain-relieving effect on PD pain [22].

In our study, participants of the IG completed the Borg scale for a subjective evaluation of how physically demanding they considered this Aviva exercise intervention. Our research has not shown that the more strenuous the Aviva exercise intervention, the greater the pain relief. The first six exercises were warm-up exercises performed in a standing position; the subsequent six included two exercises performed while sitting, with a special focus on the lower abdomen/lesser pelvis, hip, and gluteal muscles. The final six sets included one exercise performed in a lying position, besides those performed standing as part of the cooling down period [22,23,24,25]. The availability of the study coordinator and Aviva instructors for personal consultation at any time for participants in the IG, and the availability of the study coordinator for personal consultation at any time for the participants in the CG could have manifested as non-specific psychotherapeutic effects such as Hawthorne bias [41,54,55].

The power analysis (alpha = 0.05; 1 − β = 0.80) demonstrated that, due to its strong effect size, the effect of the Aviva exercise could be proven using a sample of 20 people from both groups. Therefore, in our study, the non-specific psychotherapeutic effects in both groups were surpassed.

In the Introduction, we mentioned the reliability analysis of the BAQ-H among Hungarian yoga practitioners and young adult controls carried out by Köteles in 2014. Our reliability analysis of BAQ-H was not a secondary outcome, but rather, we used Cronbach’s alpha [56] to test the reliability of BAQ-H in our study, including the participants of the Aviva-exercising IG and the CG. The Cronbach’s alpha value showed whether the abstract concepts of the four scales of the BAQ-H, e.g., “Note responses or changes in body process”, were successfully measured using simple questions. The results for the 12 Cronbach’s alpha values confirmed that we had an acceptable BAQ-H. This reliability analysis of BAQ-H was necessary for correctly using the BAQ-H in our study. The results are shown in Table S1.

The second hypothesis was not confirmed. There was no significant difference between the IG and CG regarding the different subscales of the BAQ-H at T1, T2, and T3. Only in the case of the “Note responses or changes in body process” subscale of the BAQ-H was there a trend-like effect from the Aviva exercises: the IG group is more likely to have a stronger increase than the CG group. The reduction in the level of menstrual pain between T1 and T3 can also be seen as a change in the body process. The trend between the IG and CG cannot be extrapolated to the population because of the lack of significance. On the other hand, this research highlights the fact that dysmenorrhea may affect body awareness, as interoception (visceroception) is connected with mental health [57].

Cramer and colleagues [31] sought to assess the relationship between pain-related variables and responses to interventions in patients with chronic pain using the BAQ. They concluded that body awareness and body sensitivity decreased in patients with chronic pain. Erden and colleagues [58] investigated the relationship between body awareness, quality of life, and pain and found that body awareness had a positive effect on quality of life and a negative relationship with pain.

This article is the second to explore the link between PD and body awareness. In contrast to this study, the first study [59] looked at the effects of lifestyle changes as a behavioral-based treatment and the regular use of kinesio taping. The advised lifestyle changes included reducing or avoiding smoking and drinking alcohol, the intake of caffeine, and foods high in salt; getting sufficient and quality sleep; and regular guided relaxation to reduce sympathetic activity and muscle spasms [59]. Their conclusion was that kinesio taping and lifestyle changes reduced pain severity and increased quality of life in women with PD. Furthermore, although positive results were obtained for body awareness, the changes did not reach a sufficient effect size, probably because of the short 1-month process of the study period [59]. In our study, the research period was longer, but even that was not long enough to show a significant effect on the changes in body awareness. This could be partly due to the effects of the COVID-19 pandemic, which resulted in important changes for everybody in parameters such as the fear of the disease. This had undeniable mental impacts on individuals. The high levels of stress experienced throughout the pandemic period could have suppressed bodily and mental perception. It was reported that increases in COVID-19 cases and deaths affected mental health and increased the levels of anxiety–depression in individuals [60]. Unlike our findings, the results of some studies in the existing literature show that physical activity significantly affects body awareness levels as measured using the BAQ [30,61]. Unlike in our research, in another study, not only was the BAQ used, but self-esteem, anxiety–depression levels, and quality of life were examined [61]. Even though our study’s results for BAQ-H are not statistically significant, they contribute to the literature because this study is the first to examine the connection between a mild-to-moderate exercise intervention and body awareness in women with PD. Thus, it can lead the way to further applicable studies on this topic.

There are a few other possible reasons for our second hypothesis not being confirmed. We suppose that stronger effects could have been detected in the IG if they had practiced Aviva exercises more frequently (e.g., 3–4 times weekly) in an intervention of a longer duration. The degree of change in body awareness is also related to the type and duration of the exercise intervention therapy used. Unlike the thirty-minute Aviva exercise intervention in this research, a study conducted by Daubenmier and colleagues compared the body awareness of three groups of people who performed yoga exercises for 1.5 h; 30 min of aerobic exercises, including step exercises; and no yoga or aerobic exercises or any other exercises. The results of the study showed that those who performed yoga exercises had significantly higher body awareness compared to the other groups [62]. However, body awareness is a multidimensional construct, as mentioned in the Introduction [26]. It can be modified by mental processes, including attention, attitudes and effects, appraisal, beliefs, memories, and the conditioning of the participants of the IG and the CG. The above may explain the trend-like effect found in the IG group who practiced the Aviva exercise compared to CG in our study.

There is currently no widely accepted unified and defined way to measure body awareness; besides the BAQ, other questionnaires (e.g., the Multidimensional Assessment of Interoceptive Awareness (MAIA)) can be found in the medical literature [63].

There is a need for more studies examining not only the relationship between mild-to-moderate exercises and the pain intensity in PD but also the relationships between body awareness and mild-to-moderate exercise intervention and body awareness and pain in PD, using the subscales of the BAQ. Moreover, the human personality is very complex, with components other than body awareness, such as self-esteem and anxiety–depression levels. Thus, besides BAQ, other questionnaires, e.g., the Beck Depression Scale (BDS), State and Trait Anxiety Scale (STAI), and Rosenberg Self-Esteem Scale (RSES), should be used to assess the effect of the exercise intervention in individuals with PD. Future research should also focus on the clinical implications of the exercise intervention and the seldom-provided advice for lifestyle changes to increase healthy quality of life in women with PD, as measured using tools such as the SF-36 questionnaire or Nottingham Health Profile (NHP). Furthermore, randomized controlled trials are required to assess the effects of exercise interventions and NSAIDs on pain in individuals with PD.

### Strengths and Limitations

A strength of this study is the assessment of the effects of the mild-to-moderate Aviva exercise intervention on consecutive menstrual pain in women with PD and body awareness for the first time.

Another strength of this study is that the Aviva exercise program is achievable even by individuals with a lack of free time, which is generally a characteristic of the modern lifestyle. Another strength is the flexible and realistic research, not only with adolescents but also women of all ages in the reproductive life stage who volunteered to participate in this study. Most research on PD only focuses on teenagers and young women.

The limitations of our study include the short detection period, the relatively small number of patients, and the study design of the prospective observational trial. Self-selection was also a limitation because it removed all the benefits of randomization, such as balancing confounding factors. Another limitation of this study, as a detection bias, was that only those primary dysmenorrhea patients with mild-to-moderate menstrual pain who did not take any painkillers were included in this study. For ethical reasons, we did not want to discourage any women from taking painkillers for the sake of this study who had mild, moderate, or severe menstrual pain.

This study had an allocation bias. Eight participants (20%) of the IG spontaneously reclassified themselves into the CG. Despite signing up for the IG and agreeing to take part in the Aviva exercise intervention twice a week, they did not fulfill this condition.

This dropout rate is greater than the 15% that the PEDro Scale [64] considers ideal, and the change in the allocation for 20% of the participants should be considered.

## 5. Conclusions

Our study is the first to document the effects of the mild-to-moderate Aviva exercise intervention on consecutive pain levels of different menstrual cycles and body awareness in women with PD. In our study, Aviva exercises could contribute to pain relief from PD. Regarding body awareness, no significant difference was found between the IG and CG, although a trend-like effect from the Aviva exercises was found. Due to the limitations of our study, the short detection period, the prospective observational design, and the multidimensional complexity of the human personality, further research is warranted to verify these results.

## Figures and Tables

**Table 1:** Distribution of demographic data and dysmenorrhea scores (NRS) between the two groups at baseline.

			K-S *					LTfEoV ****		*t*-Test	
Variables	Group	n	D	*p*	Me **	Mean	S. D. ***	F	*p*	t	*p*
Dysmenorrhea scores	CG	35	0.094	0.200	2.550	2.499	1.336	0.253	0.616	0.754	0.453
	IG	40	0.126	0.200	2.000	2.270	1.295				
Age distribution (year)	CG	38	0.101	0.200	34.000	33.605	6.258	0.000	0.997	0.521	0.604
	IG	40	0.098	0.200	33.000	32.875	6.115				
BMI	CG	38	0.132	0.157	22.315	23.389	4.812	2.399	0.126	1.682	0.097
	IG	40	0.115	0.200	20.964	21.763	3.675			M-W *****	
										z	*p*
Weight (kg)	CG	38	0.124	0.200	63.000	65.158	13.060	0.733	0.395	−1.901	0.057
	IG	40	0.183	0.017	55.500	59.525	10.505				
Duration of the first menstrual cycle (day)	CG	38	0.264	<0.001	28.000	30.500	9.058	1.916	0.170	1.394	0.167
	IG	40	0.171	0.035	28.000	28.350	3.541				
Duration of the second menstrual cycle (day)	CG	37	0.230	<0.001	29.000	31.243	6.238	3.956	0.050	−1.038	0.299
	IG	38	0.158	0.071	29.000	29.237	4.365				
Age at onset of menstruation (year)	CG	35	0.171	0.015	13.000	12.829	1.445	0.450	0.505	−1.032	0.302
	IG	35	0.199	0.006	13.000	13.171	1.317				
Age at the first dysmenorrhea (year)	CG	33	0.208	0.001	14.000	15.515	5.292	0.205	0.652	−1.017	0.309
	IG	29	0.247	<0.001	15.000	15.966	4.040				
Number of births	CG	35	0.485	<0.001	0.000	0.257	0.561	0.799	0.375	−1.256	0.209
	IG	35	0.539	<0.001	0.000	0.171	0.618				

* Kolmogorov–Smirnov test ** Median *** Standard deviation **** Levene’s test for equality of variances ***** Mann–Whitney test.

**Table 2:** Test results of repeated-measure ANCOVA analysis of menstrual pain change.

Source	Type III Sum of Squares	df	Mean Square	F	*p*	Partial Eta Squared
Menst. Pain CHG ^a^	0.029	1.607	0.018	0.027	0.951	<0.001
Menst. Pain CHG × Age	0.270	1.607	0.168	0.257	0.725	0.003
Menst. Pain CHG × BMI	0.730	1.607	0.454	0.694	0.471	0.009
Menst. Pain CHG × Aviva ^b^	13.393	1.607	8.335	12.743	<0.001	0.147
Error (Menst. Pain CHG)	77.771	118.907	0.654			

^a^ Menstrual pain change ^b^ Aviva exercise.

**Table 3:** Estimated marginal means of menstrual pain of the CG and IG at T1, T2, and T3.

ToM *	CG			IG		
	n	Mean	SD **	n	Mean	SD
T1	35	2.43	1.325298	40	2.33	1.291325
T2	35	2.42	1.498123	40	2.01	1.351697
T3	35	3.25	1.649221	40	1.00	0.734987

* Time of measurement ** Standard deviation (*p* < 0.001).

**Table 4:** Test results of repeated measures ANCOVA of BAQ-H.

		Type III					
Scale	Source	Sum of Squares	df	Mean Square	F	*p*	Partial Eta Squared
Note responses or changes in body process	BAQ-H	0.331	1.823	0.182	0.824	0.431	0.011
	BAQ-H × Age	0.899	1.823	0.493	2.235	0.116	0.031
	BAQ-H × BMI	0.338	1.823	0.186	0.841	0.424	0.012
	BAQ-H × AVIVA	1.033	1.823	0.567	2.569	0.086	0.035
	Error (BAQ-H)	28.557	129.401	0.221			
Predict of bodily reaction	BAQ-H	0.024	1.845	0.013	0.064	0.926	0.001
	BAQ-H × Age	0.549	1.845	0.298	1.488	0.230	0.021
	BAQ-H × BMI	1.654	1.845	0.897	4.483	0.015	0.059
	BAQ-H × AVIVA	0.339	1.845	0.184	0.920	0.394	0.013
	Error (BAQ-H)	26.193	130.970	0.200			
Sleep-wake cycle	BAQ-H	0.350	1.919	0.182	1.329	0.268	0.018
	BAQ-H × Age	0.002	1.919	0.001	0.008	0.991	0.000
	BAQ-H × BMI	1.143	1.919	0.595	4.340	0.016	0.058
	BAQ-H × AVIVA	0.573	1.919	0.299	2.177	0.119	0.030
	Error (BAQ-H)	18.698	136.274	0.137			
Onset of illness	BAQ-H	0.174	1.692	0.103	0.289	0.712	0.004
	BAQ-H × Age	0.262	1.692	0.155	0.434	0.615	0.006
	BAQ-H × BMI	0.162	1.692	0.096	0.268	0.728	0.004
	BAQ-H × AVIVA	0.665	1.692	0.393	1.104	0.327	0.015
	Error (BAQ-H)	42.788	120.102	0.356			

**Table 5:** A comparison of the means of the four scales of the BAQ-H of the CG and IG at T1, T2, and T3.

	Note Responses or Changes in Body Process **		Predict of Bodily Reaction		Sleep-Wake Cycle		Onset of Illness	
ToM *	CG	IG	CG	IG	CG	IG	CG	IG
T1	4.98	5.10	5.11	5.09	5.29	5.23	5.11	5.09
T2	5.19	5.35	5.20	5.33	5.40	5.41	5.20	5.33
T3	5.20	5.60	5.26	5.53	5.40	5.60	5.26	5.53

* Time of measurement ** *p* = 0.086.

## Data Availability

The data used to support the findings of this study are available from the corresponding author upon request.

## Supplementary Materials

The following supporting information can be downloaded at: https://www.mdpi.com/article/10.3390/medicina60010184/s1, Figure S1: The Aviva Exercise Manual. Table S1: Test results of repeated-measure ANCOVA of BAQ-H.
