# Supplementary material for: The Effect of Aviva Exercise Intervention on Pain Level and Body Awareness in Women with Primary Dysmenorrhea
Source: Medicina (Kaunas). 2024 Jan 20;60(1):184. doi: 10.3390/medicina60010184 (PMC10821191; doi:10.3390/medicina60010184)
Supplement: Supplementary file 1 [file medicina-60-00184-s001.zip › Figure S1..pdf]

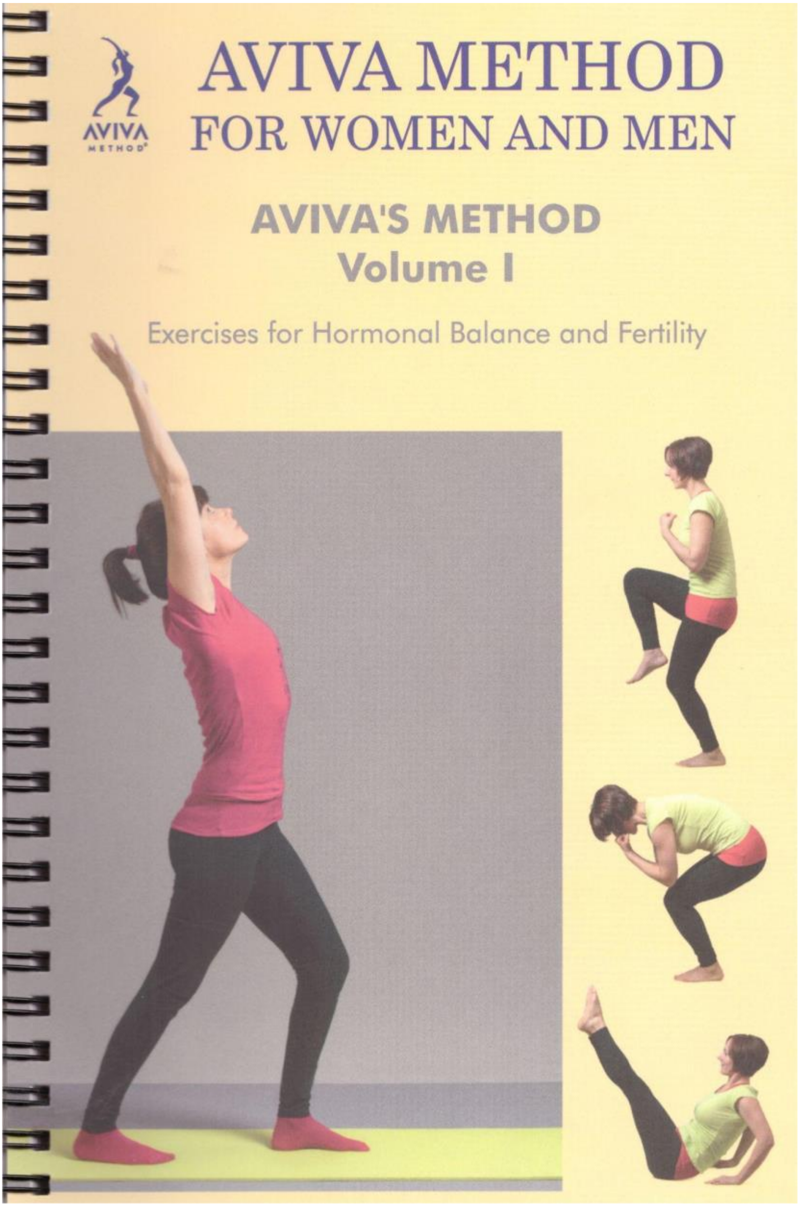

AVIVA SIMPLE METHOD
Manual

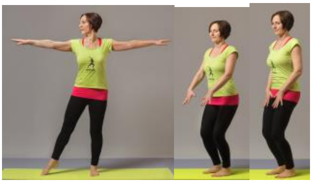	**1. Chassé**<br><br>**A) 4x**—4 chassé-steps, 1 tap (moving back and forth, right–left)<br>**B) 4x**—3 chassé-steps, turn, tap (moving right–left)<br>**C) 6x**—2 chassé-steps, turn, tap, continue same direction 2 chassé-steps, turn, tap (moving right–left)
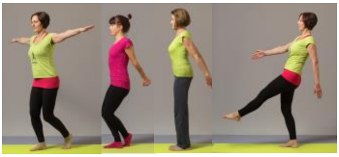	**2. "ZORBA"**<br><br>**A) 3x—Zorba**: step forward, contract WZ (working zone); step back, contract WZ; step to the side, contract WZ; step back, contract WZ<br>**B) 36x—Syrtaki**: cross step + hip tilt, step to the side + relax<br>**C) 2x**—16 **arm rotation** forward, 16 to the side, 16 behind your back<br>**D) 4x—Fast Zorba**<br>**E) 4x—Rag Doll**: forward, backward, side to side
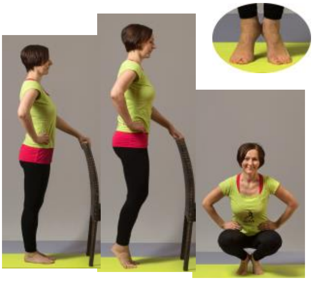	**3. Next to Chair on Tiptoes**<br><br>**A) 8x**—Rising to the balls of your feet, contract, descend to your feet and relax<br>**B) 4x**—Rising to the balls of your feet, contract for a count of 8, descend to your feet and relax<br>**C) 8x**—Rising to the balls of your feet, contract, then drop back on your heels<br>**D) 4x**—Rising to the balls of your feet, contract, squat down, stand up, lower your heels, relax
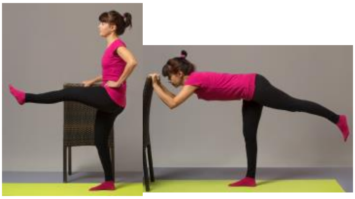	**4. LEG SWINGS**<br><br>**A) 8x**—Swing your leg forward and contract WZ<br>**B) 8x**—Lift your leg to the side<br>**C) 8x**—Bend forward and swing your leg backwards; your hips remain square facing the ground

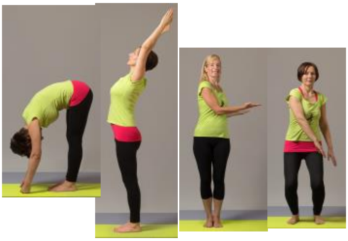	**5. BOWING AND RELAXING**<br>**A) 3x**—Bend forward, come up and lean back with hands up<br>**B) 4x**—Shoulders and arms twist right and left (head and hips facing forward)<br>**C) 4x**—Relaxation, arms swing left and right; while bouncing from your knees, your whole torso twists
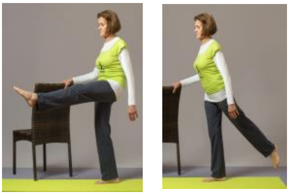	**6. LEG SWING**<br><br>**A) 16x**—Swing your right leg back and forth, legs are straight<br>**B) 16x**—Swing your left leg back and forth, legs are straight
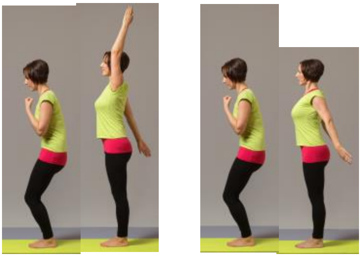	**7. RESTORING EXERCISE**<br><br>**A) 16x**—Tilt your hips and contract WZ, then relax and swing one arm up and the other arm down<br>**B) 16x**—Tilt your hips and contract WZ, then relax and swing both arms behind your back
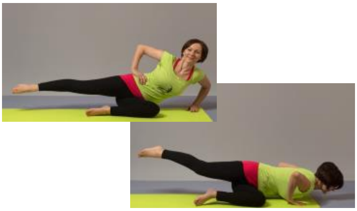	**8. “Z” SITTING**<br><br>**A) 4x**—Stretched out leg pulls back, then bring it to the side, lengthen; switch legs and repeat the exercise<br>**B) 4x**—Stretched out leg pulls back, then bring it to the side, twist your torso; switch legs and repeat the exercise

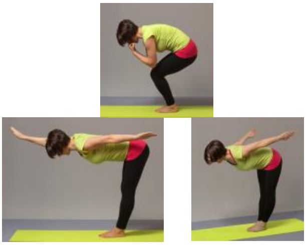

9. PUMP AND ROCKET

A) 16x—Pump: lean forward, contract WZ, then straighten your knees and relax, one arm swings forward and one arm swings back
B) 16x—Rocket: lean forward, contract WZ, then straighten your knees and relax, swing both arms backwards at the same time

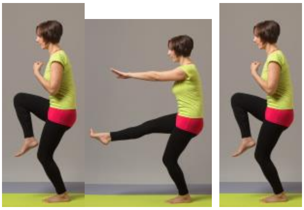

10. SPRING

A) 8x—Pull knee to chest, alternate legs
B) 16x—Pull knee to chest, kick, pull knee up again and alternate legs
C) 16x—Pull knee to chest, kick, pull knee to chest again then step forward

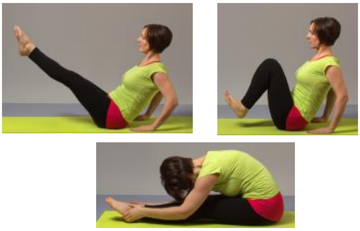

11. FOOTBALL

A) 4x—Flex your feet and stretch your legs forward, then lift your legs up 45 degrees, and pull your knees to your chest
B) 4x—Flex your feet and stretch your legs forward, then lift your legs 45 degrees, hold position while counting to 4, then pull your knees to your chest
C) 1x—Lean forward onto your straight legs

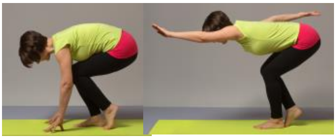

12. MONKEY WALK

A) 16x—Little Monkey: bounce deeply 2x; your bottom is moving up and down, your arms loosely hang down
B) 16x—Big Monkey: bounce deeply 2x; your bottom is moving up and down, one arm swings forward and the other arm backwards, alternate your arms as you move

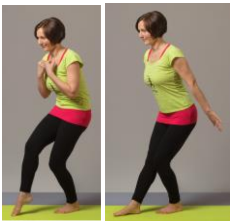

13. LITTLE STEP

16x—*Right* leg stretches forward, bend your knee and tilt your pelvis, contract WZ, then straighten your leg, relax WZ (left leg stays bent)
16x—*Left* leg stretches forward, bend your knee and tilt your pelvis, contract WZ, then straighten your leg, relax WZ (right leg stays bent)

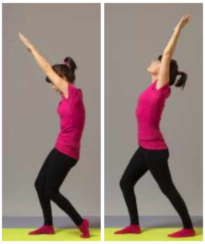

14. THE SYMBOL EXERCISE

16x—***Right*** *leg* is forward and bent, bend your left knee and tilt your pelvis, contract WZ, then stretch out your left knee, relax WZ (right leg stays bent for the entire time)
16x—***Left*** leg forward (repeat movements on the left side**)**

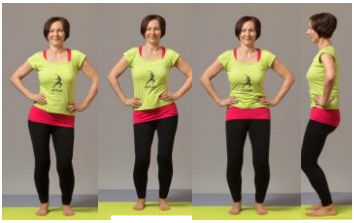

15. 1, 2, BUMM

4x—1, 2, Bumm: tilt your hips to the right then to the left, then tilt your hips forward and contract WZ
8x—Tilt your hips side to side
4x—1, 2, Bumm
8x—Tilt your hips side to side
4x—1, 2, Bumm

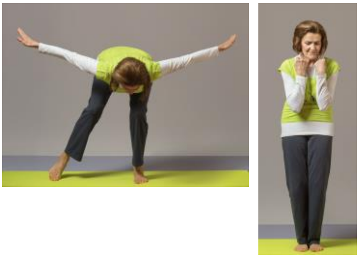

16. BIRD-OF-PARADISE

A) 16x—Lean forward and contract WZ, stomp with your right leg then your left leg to the side while relaxing WZ
B) 16x—Straighten up and contract WZ, stomp with your right leg then your left leg to the side while you relax WZ
Repeat the movement while circling around
C) 16x—Lean forward and contract WZ, circle around while stomping out to the side first with right leg then with left leg, when stepping out, relax WZ
D) 8x—Straighten up and repeat movements with easy and light jumps while circling around one direction then the other

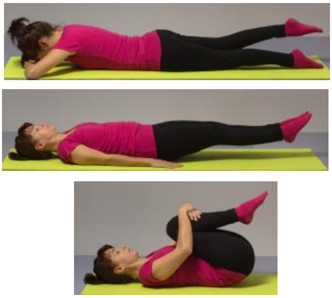

17. PRONE

A) 4x—Lie face down, squeeze your glutes, lift your right leg
1x—Hold it for a count of 4
Repeat the exercise with left leg
Repeat the exercise with both legs together
B) 4x—Lie on your back, stretch your legs out toward the ceiling, lower your legs and stop at 45 degrees to hold, then lower legs again, stop 5 cm from the floor and drop your heels with a thud
C) 4x—Lie on your back, hug your knees with your hands to your chest and squeeze your glutes, then relax

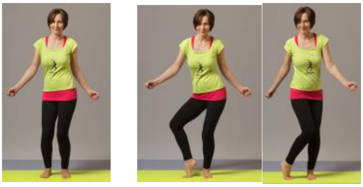	**18. TOWEL EXERCISE**<br><br>**A) 16x**—Turn your hips right and left (knees bent)<br>**B) 32x**—Ball of your foot presses down, twist your leg from the hip socket<br>**C) 32x**—Step forward, turning in a circle, your foot stays anchored, pushing the ball of your foot firmly down, your leg and the respective hip twist simultaneously
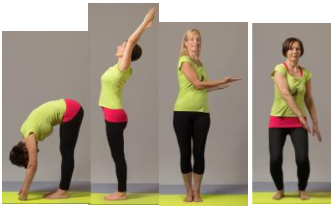	**19. BOWING AND RELAXING**<br><br>**A) 3x**—Bend forward, come up and lean back with hands up<br>**B) 4x**—Shoulders and arms twist right and left (head and hips facing forward)<br>**C) 4x**—Relaxation, arms swing left and right; while bouncing from your knees, your whole torso twists

For your own health benefit only sign up for workshops with OFFICIALLY CERTIFIED AVIVA METHOD TRAINER: www.avivamodszer.hu. It is important to perform the exercises precisely and regularly in order to reach the desired therapeutic effect of the method.

ATTENTION!

Please note that the materials above are only a guide to help you learn the Aviva Method. You may use this manual after the official workshop, but in order to more thoroughly learn the Method, the guide by itself is not enough. The exercises will be effective only if they are performed correctly and regularly. The Aviva Method trainer can always customize the exercises to your individual needs. The exercises are performed at your own risk based on the information you received during the Aviva Method Workshop and on the following of the instructions of the Aviva Method Book.

The list of certified Aviva Method instructors can be found here: http://avivamodszer.hu

Aviva Foundation, E-mail: info@avivamodszer.hu

Photographs by the Aviva Foundation, with the aid of professional Aviva Method instructors.

Aviva word mark in Hungary: Registration Number: 222245

Aviva Method figurative mark EU:

Registration Number : 012815651

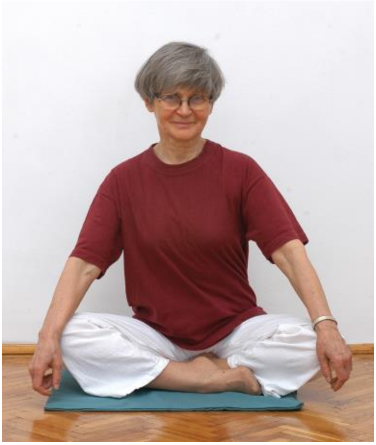

All rights reserved!
No parts of this current publication can be reproduced or made public without prior permission from the publisher.
