# Supplementary material for: The Effect of Aviva Exercise Intervention on Pain Level and Body Awareness in Women with Primary Dysmenorrhea
Source: Medicina (Kaunas). 2024 Jan 20;60(1):184. doi: 10.3390/medicina60010184 (PMC10821191; doi:10.3390/medicina60010184)
Supplement: Supplementary file 1 [file medicina-60-00184-s001.zip › Table S1.pdf]

Table S1. Descriptive and reliability statistics of the four subscales of the BAQ-H at the T1, T2 and T3

Scale	Items	n	Min*	Max**	Mean	Std. Dev. ***	Cronbach's alpha
Note responses or changes in body process – T1	5	78	3.00	6.80	5.02	0.92	0.708
Predict of bodily reaction – T1	7	78	2.29	7.00	4.68	0.90	0.759
Sleep-wake cycle – T1	7	78	3.00	6.57	5.26	0.80	0.734
Onset of illness – T1	3	78	2.00	7.00	5.11	0.92	0.453
Note responses or changes in body process – T2	5	78	3.40	7.00	5.27	0.86	0.716
Predict of bodily reaction – T2	7	78	2.29	6.71	4.90	0.90	0.808
Sleep-wake cycle – T2	7	78	2.86	7.00	5.39	0.81	0.798
Onset of illness – T2	3	78	3.00	7.00	5.26	0.91	0.432
Note responses or changes in body process – T3	5	75	3.80	7.00	5.40	0.75	0.657
Predict of bodily reaction – T3	7	75	3.00	7.00	5.05	0.86	0.836
Sleep-wake cycle – T3	7	75	3.71	7.00	5.50	0.75	0.813
Onset of illness – T3	3	75	2.67	7.00	5.40	0.91	0.637

* Minimum ** Maximum *** Standard deviation
